# Dataset of observables for small modular lead-cooled fast reactor MOX spent nuclear fuel

**DOI:** 10.1016/j.dib.2025.111513

**Published:** 2025-03-22

**Authors:** Victor J. Casas-Molina, Pablo Romojaro, Gwennaelle Nourry, Gert Van den Eynde, Luca Fiorito, Ivan Merino-Rodríguez, Tom Dhaene, Ivo Couckuyt

**Affiliations:** a SCK CEN, Belgian Nuclear Research Centre, Boeretang 200, Mol 2400, Belgium; b Department of Information Technology, Ghent University-imec, Technologiepark-Zwijnaarde 126, Ghent 9052, Belgium; c Conservatoire National des Arts et Métiers, Rue Saint-Martin 292, Paris 75003, France; d Universidad Católica del Maule, Talca 3460000, Chile

**Keywords:** Spent nuclear fuel, MOX, SMR, LFR, Nuclide inventory, Decay heat, Radiotoxicity, Burnup

## Abstract

Lead-cooled Fast Reactors (LFRs) with mixed-oxide (MOX) fuel are promising candidates in the Generation IV (Gen IV) small modular reactor (SMR) landscape due to their capacity for actinides transmutation, passive safety features, and minimized waste radiotoxicity. For secure management, storage, safeguards, rigorous characterization is necessary. This database was developed to support the optioneering and design of MOX-based lead-cooled fast reactors. This data article introduces a comprehensive dataset of isotopic mass densities, spanning 152 nuclides present in irradiated LFR-MOX fuel, additionally providing insights into fuel characteristics such as activity, decay heat rates, photon emission rates, spontaneous fission rates, and radiotoxicity values across various decay steps.

Using the Serpent2 Monte Carlo code for fuel depletion calculations, and processed with SerpentTools, the dataset captures inventory data as a function of reactor power, fuel burnup, plutonium vector in the fresh MOX, and decay time at the end of irradiation, enabling analyses of SNF properties. The dataset is stored in Parquet format, including one primary depletion file and 13 decay files.

Specifications Table

**Table:** 

Subject	*Nuclear Energy and Engineering*
Specific subject area	*Nuclear spent fuel characterization, nuclear safeguards and fuel cycle analysis for lead-cooled fast reactors (LFRs)*
Type of data	Table, raw, filtered, processed
Data collection	Data were extracted from the depletion output files from Serpent2 calculations with SerpentTools package and then processed using Pandas
Data source location	*Institution: Belgian Nuclear Research Centre (SCK CEN)* *City: Mol* *Country: Belgium*
Data accessibility	Repository name: Mendeley Data Data identification number: 10.17632/9gsz2cddwg.1 Direct URL to data: https://data.mendeley.com/datasets/9gsz2cddwg/1
Related research article	None

## 1 Value of the Data

• The database contains information for the study of mixed oxide (MOX) fuel cycle in Lead-cooled Fast Reactors (LFR) for Small Modular Reactor (SMR) power scales. The dataset contains the inventory evolution over time of 152 nuclides, selected as observables for their importance related to Spent Nuclear Fuel (SNF) characterization (burnup indicators, decay heat contributors, and neutron and gamma emitters), fuel cycle analysis (heavy metal and fission product masses, radiotoxicity) and nuclear safeguards (fissile content).
• Additionally, activity, decay heat rates, spontaneous fission rates, (α,n) neutron emission rates, photon emission rates, and radiotoxicity have been calculated for different decay times on the spent fuel; and for different plutonium isotope vector proportions in the fresh fuel, Initial Plutonium Content (IPC) and discharge burnup, i.e. the burnup reached at the end of the fuel residence time in the core, which supplements the nuclide mass inventory information.
• The dataset can contribute to the research on the fuel cycle for the next generation of SMRs based on the LFR concept, for which to the best of our knowledge, there are not yet developed robust benchmarks on the depletion and the characterization of the fuel at reactor discharge. This database saves time and resource-consuming calculations, that are required when using fuel depletion codes based on precomputed libraries.
• The present dataset complements the previously published libraries in Refs. [[1], [2], [3]], in which similar datasets for Light Water Reactor SNF obtained with SCALE6.1 [4] and Serpent2 [5] codes were published, aiming to contribute to a robust dataset presence regarding SNF cycle research.

## 2 Background

Since the early days of nuclear power, small and medium-sized reactors have been considered. However, the trend leaned towards larger units due to economies of scale. Recently, Small Modular Reactors have gained appeal [6].

Currently, over 80 SMR concepts are in development, based on thermal and fast neutron spectra, using different fuel and coolant technologies. Among Gen IV SMRs, LFRs stand out for their passive safety features, such as chemical inertness and natural circulation cooling. Lead coolant allows operation in the fast-neutron spectrum, aiding in plutonium breeding or actinide burning, thus reducing waste volume and radiotoxicity [7].

Due to the presence of radionuclides, a SNF assembly needs to be characterized, i.e., certain characteristics have to be studied for a safe, secure, ecological and economical transport, intermediate storage and final disposal. Some of these observables can be measured, however this is often complex, time-consuming and expensive. Therefore, full characterization of a SNF usually relies on theoretical model calculations with core simulator or fuel depletion codes [8].

## 3 Data Description

The dataset consists of two directories; the first one has the simulations with no decay, *SCKCEN-MOX-LFR-SMR*. It contains a columnar format Parquet file (*SCKCEN-MOX-LFR-SMR.parquet*), which includes the information related to MOX fuel burnup process and it is structured in a matrix with a size of 960,000 (rows) x 161 (columns). The first column is ‘POWER’ and indicates the reactor linear power density in W/cm, the second column is ‘BU’ which contains the discharge burnup values for the fuel in MWd/kg_HM_ (HM stands for initial Heavy Metal: U, Pu and Am content); the third column is named ‘IPC’ and indicates the initial plutonium content^1^ for the fresh fuel in percentage taken over the heavy metal mass. The next six columns contain the initial normalized Pu vector proportions in % in weight in the fuel, namely: ‘Pu238_IPC’, ‘Pu239_IPC’, ‘Pu240_IPC’, ‘Pu241_IPC’, ‘Pu242_IPC’ and ‘Am241_IPC’. The next 152 columns contain the output of the simulation: mass density in g/cm^3^ of the nuclides:

‘*H3*’, ‘*He4*’, ‘*O16*’, ‘*Se79*’, ‘Se80’, ‘Se82’, ‘Br81’, ‘Kr81’, ‘Kr83’, ‘Kr84’, ‘Kr85’, ‘Kr86’, ‘Rb85’, ‘Rb87’, ‘Sr88’, ‘***Sr90***’, ‘Y89’, ‘***Y90***’, ‘Zr90’, ‘Zr91’, ‘Zr92’, ‘*Zr93*’, ‘Zr94’, ’Zr95’, ‘Zr96’, ‘Nb93m’,’ Nb95’, ‘Mo95’, ‘Mo96’, ‘Mo97’, ‘Mo98’, ‘Mo100’, ‘Tc99’, ‘Ru100’, ‘Ru101’, ‘Ru102’, ‘Ru104’, ‘***Ru106***’, ‘Rh103’, ‘**Rh106**’, ‘Pd104’, ‘Pd105’, ‘Pd106’, ‘Pd107’, ‘Pd108’, ‘Pd110’, ‘Ag109’, ‘Cd110’, ‘Cd111’, ‘Cd112’, ‘Cd113m‘, ‘Cd114’, ‘Cd116’, ‘Sn116’, ‘Sn117’, ‘Sn118’, ‘Sn119’, ‘Sn120’, ‘Sn122’, ‘Sn124’, ‘*Sn126*’, ‘Sb121’, ‘Sb123’, ‘*Sb125’*, ‘*Te125*’, ‘Te125m’, ‘Te128’, ‘Te130’, ‘I127’, ‘I129’, ‘Xe128’, ‘Xe130’, ‘Xe131’, ‘Xe132’, ‘*Xe134*’, ‘Xe136’, ‘**Cs133**’, ‘**Cs134**’, ‘Cs135’, ‘***Cs137***’, ‘Ba134’, ‘Ba136’, ‘Ba137’, ‘**Ba137m**’, ‘Ba138’, ‘La139’, ‘*Ce140*’, ‘Ce142’, ‘**Ce144**’, ‘Pr141’, ‘Nd142’, ‘Nd143’, ‘Nd144’, ‘Nd145’, *‘Nd146*’, ‘**Nd148**’, ‘Nd150’, ‘Pm147’, ‘Sm146’, ‘Sm147’, ‘Sm148’, ‘**Sm149**’, ‘Sm150’, ‘Sm151’, ‘Sm152’, ‘Sm154’, ‘Eu153’, ‘**Eu154**’, ‘Eu155’, ‘Gd156’, ‘Gd158’, ‘Tb159’, ‘Ho166m’, ‘*Pb206*’, ‘*Pb207*’, ‘*Pb208*’, ‘*Pb210*’, ‘*Bi209*’, ‘Ac227’, ‘Th228’, ‘Th229’, ‘Th230’, ‘Th232’, ‘Pa231’, ‘*U232*’, ‘*U233*’, ‘*U234*’, ‘*U235*’, ‘*U236*’, ‘*U238*’, ‘*Np237*’, ‘***Pu238***’, ‘***Pu239***’, ‘***Pu240***’, ‘***Pu241***’, ‘*Pu242*’, ‘*Pu244*’, ‘***Am241***’, ‘*Am242m*’, ‘*Am243*’, ‘*Cm242*’, ‘*Cm243*’, ‘***Cm244***’, ‘*Cm245*’, ‘*Cm246*’, ‘*Cm247*’, ‘*Cm248’*, ‘*Cm250*’, ‘*Bk249*’, ‘*Cf249’*, ‘*Cf250*’, ‘*Cf251*’.

These nuclides were selected based on Refs. [8,9]. If in the above list a nuclide appears in bold, it is because it has been added due to the justification from [8]; if it is in italic, it has been found relevant because of [9] and if it is both bold and italic, it was mentioned in both Refs. The remaining nuclides are the contributors added for mass conservation in fuel cycle calculations, e.g., the ^16^O, which constitutes an important fraction of the mass in the SNF.

The nuclides are named by their chemical symbol and atomic mass number. If an ‘m’ is present after the atomic mass number, it means that the isotope is in a metastable state (‘Nb93m’, ‘Cd113m’, ‘Te125m’, ‘Ba137m’ and ‘Ho166m’).

In the second directory there are the decay simulations, *SCKCEN-MOX-LFR-SMR-DECAY.* There are 13 Parquet files named as ‘*SCKCEN-MOX-LFR-SMR-DECAY-BU-{XXX}.parquet’*, where XXX is the discharge burnup in MWd/kg_HM_ for the samples of MOX fuel contained in the file in a range that goes from 30 MWd/kg_HM_ to 150 MWd/kg_HM_ in steps of 10 MWd/kg_HM_. Lower discharge burnups have not been provided because average fuel assembly discharge burnup greater than 30 MWd/kg_HM_ is envisioned in the majority of the SMR LFR reactor concepts available. Each file is structured as a 240,000 rows x 1073 columns matrix. These files contain the inventory evolution of the selected nuclides for the decay time elapsed since the moment the fuel reaches the target burnup, given by the first column, ‘*Time*’, measured in days. The next eight columns are: ‘POWER’, ‘IPC’, ‘Pu238_IPC’, ‘Pu239_IPC’, ‘Pu240_IPC’, ‘Pu241_IPC’, ‘Pu242_IPC’ and ‘Am241_IPC’; the next 152 columns contain the mass density in g/cm^3^ of the observed nuclides that were listed above.

The remaining columns contain activity, decay heat rates, spontaneous fission rates, (α,n) neutron emission rates, photon emission rates and radiotoxicity of each of the 152 nuclides, and have the same structure and order as the columns containing the mass density apart from a suffix in their labels. The only exception is for the columns of the (α,n) emission rate, in which only the spontaneous alpha particle emitters ^238^Pu, ^239^Pu, ^240^Pu, ^241^Am, ^242^Cm and ^244^Cm, are considered. Information about the meaning of suffixes is given in Table 1, being the order of appearance for the suffixes in the table the same as the columns order in the dataset. Note that these observables are given on a per axial length basis (in cm units), since the calculations are performed in 2D [5].

**Table 1:** Suffixes employed in the dataset. Example given for Pu239.

‘Pu239_A’	Activity in Becquerels per cm, of ^239^Pu
‘Pu239_H’	Decay Heat in Watts per cm, of ^239^Pu
‘Pu239_SF’	Spontaneous fission rate in fissions per second per cm, of ^239^Pu
‘Pu239_AN’	(α,n) emission rate in neutrons per second per cm, of ^239^Pu
‘Pu239_GSRC’	Photon emission rate in photons per second per cm, of ^239^Pu
‘Pu239_ING_TOX’	Ingestion toxicity in Sieverts per cm, of ^239^Pu^1^
‘Pu239_INH_TOX’	Inhalation toxicity in Sieverts per cm, of ^239^Pu

1 The conversion factors used were the default values in Serpent2, which are obtained from [10].

For handling the data, the pandas package for Python [11] can be used as:

import pandas as pd

df_pandas = pd.read_parquet('SCKCEN-MOX-LFR-SMR.parquet)

If the handling using pandas is too heavy for certain applications, a more lightweight option such as polars package can be used [12]:

import polars as pl

df_polars = pl.read_parquet('SCKCEN-MOX-LFR-SMR.parquet')

As an estimate, the polars package is five times quicker loading the data frame.

## 4 Experimental Design, Materials and Methods

### 4.1 Experimental design

The span of the design variables is such that it covers typical composition ranges for the fuel and normal operational states for the reactors. The design variables include: POWER, BU, IPC, DECAY TIME, ‘Pu238_IPC’, ‘Pu239_IPC’, ‘Pu240_IPC’, ‘Pu241_IPC’, ‘Pu242_IPC’, and ‘Am241_IPC’.

The main objective of the experimental design was to provide observations from the system variables at intervals as regular as possible, allowing the end user to perform simple interpolation with acceptable accuracy. Some design variables—namely, POWER, BU, and IPC—were taken at regular intervals within their predefined ranges to achieve this. DECAY TIME, however, was an exception. Its steps were fixed at 1, 5, 10, 30, 100, 1000, 10,000, 100,000, and 1,000,000 years, since the evolution of decay is exponential, and a linear discretization would not be very informative. Nonetheless, decay calculations are computationally less demanding, and if desired, they can be recalculated for any necessary value using simple numerical methods.

Once the intervals were defined, the space described by the previous variables was generated by following a Full Factorial Design, meaning that all possible combinations of the defined variable levels were systematically explored to ensure comprehensive coverage of the parameter space.

Later, for modeling the MOX composition, and due to the computational cost of the simulations, the plutonium vector: ^238^Pu, ^239^Pu, ^240^Pu, ^241^Pu, ^242^Pu proportions (e.g., proportion of ^239^Pu is ^239^Pu/(Pu+^241^Am), was sampled 500 times using a Multivariate Normal (MVN) distribution. The MVN distribution was chosen to represent the correlations between isotopes that result from the physical transmutation process occurring in Pressurized Water Reactors (PWRs), which is more informative than assuming a uniform distribution.

**Fig. 1:**
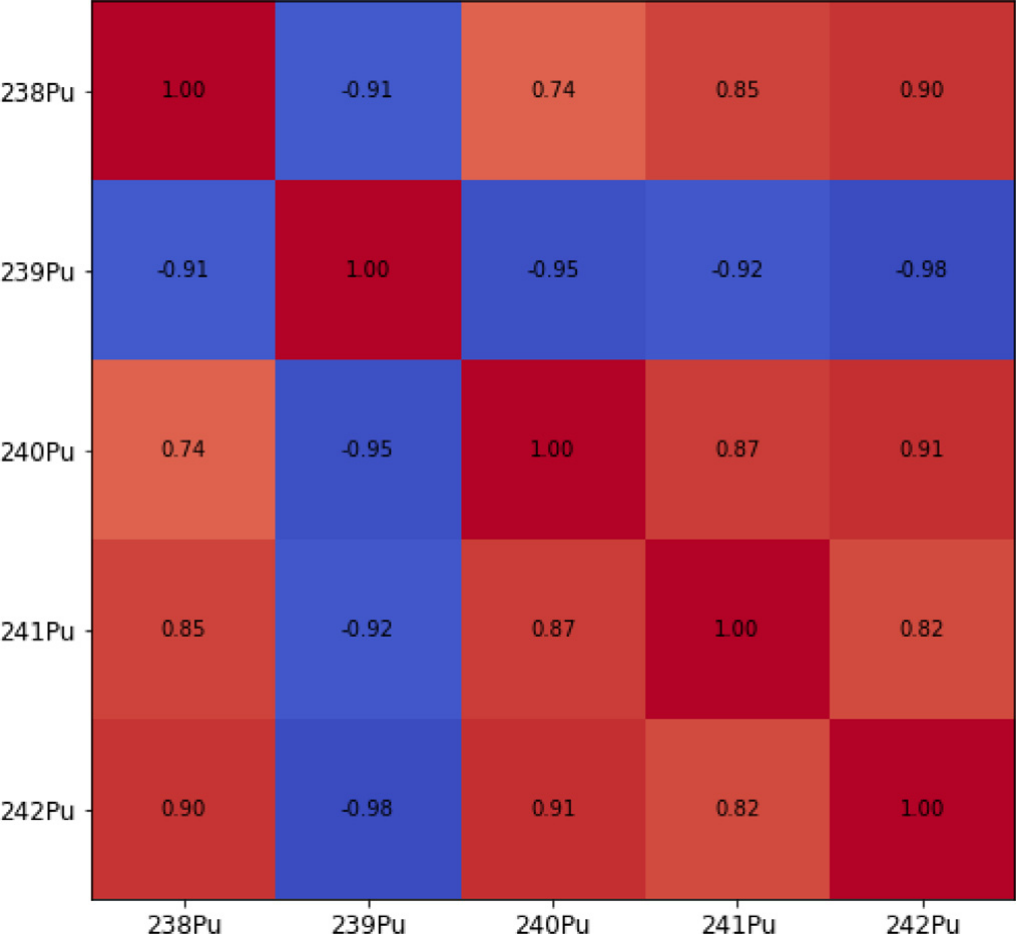
Sample correlation matrix from SCK CEN sampled database [1].

The MVN distribution originated from the covariances from those nuclides present in an SNF database generated at SCK CEN for fresh PWR UOX fuel [1]. This approach reflects the relation between isotopes found in SNF from PWRs, resulting from transmutation processes that govern the production chain. PWR SNF is the base material used in reprocessing^2^ to produce MOX fuel, assuming no other source of plutonium. Therefore, the correlations present between isotopes in the SNF must be representative to those found in freshly reprocessed base material for MOX fabrication. Only the UOX SNF with an initial ^235^U enrichment larger than 3% and a discharge burnup above 30 MWd/kg_HM_ was chosen, since below these values, there is not enough plutonium to economically justify the reprocessing of the UOX SNF [13,14]. The resulting sampled correlation matrix can be observed in Fig. 1.

In Table 2 the average values for the different plutonium isotopes can be found:

**Table 2:** Sample average plutonium vectors in weight %.

^238^Pu	2.93 %
^239^Pu	53.85%
^240^Pu	21.93 %
^241^Pu	14.44 %
^242^Pu	6.35 %

To model the ^241^Am presence in the reprocessed MOX, the average age of a MOX fuel assembly extracted from [15] was considered to model the decay of ^241^Pu and subsequent buildup of ^241^Am content. According to the reference, European MOX was estimated to be on average 3.4 ± 0.8 years old. It is also known that 67% of the fuel age is within ± 25% of the age average value, and that the minimum age was 1.2 years and the maximum 6.3. Therefore, the decay of ^241^Pu was modeled by applying the exponential decay law to a MOX age sampled from a truncated normal (µ=3.4 y, σ=0.8 y, min = 1.2 y, max = 6.3 y), described by the aforementioned distribution momenta. The sampled quantities were subtracted from ^241^Pu and added to ^241^Am mass contents, after that, the heavy metal mass content in the fuel was normalized. Due to computational limitations only 500 samples were drawn.

**Fig. 2:**
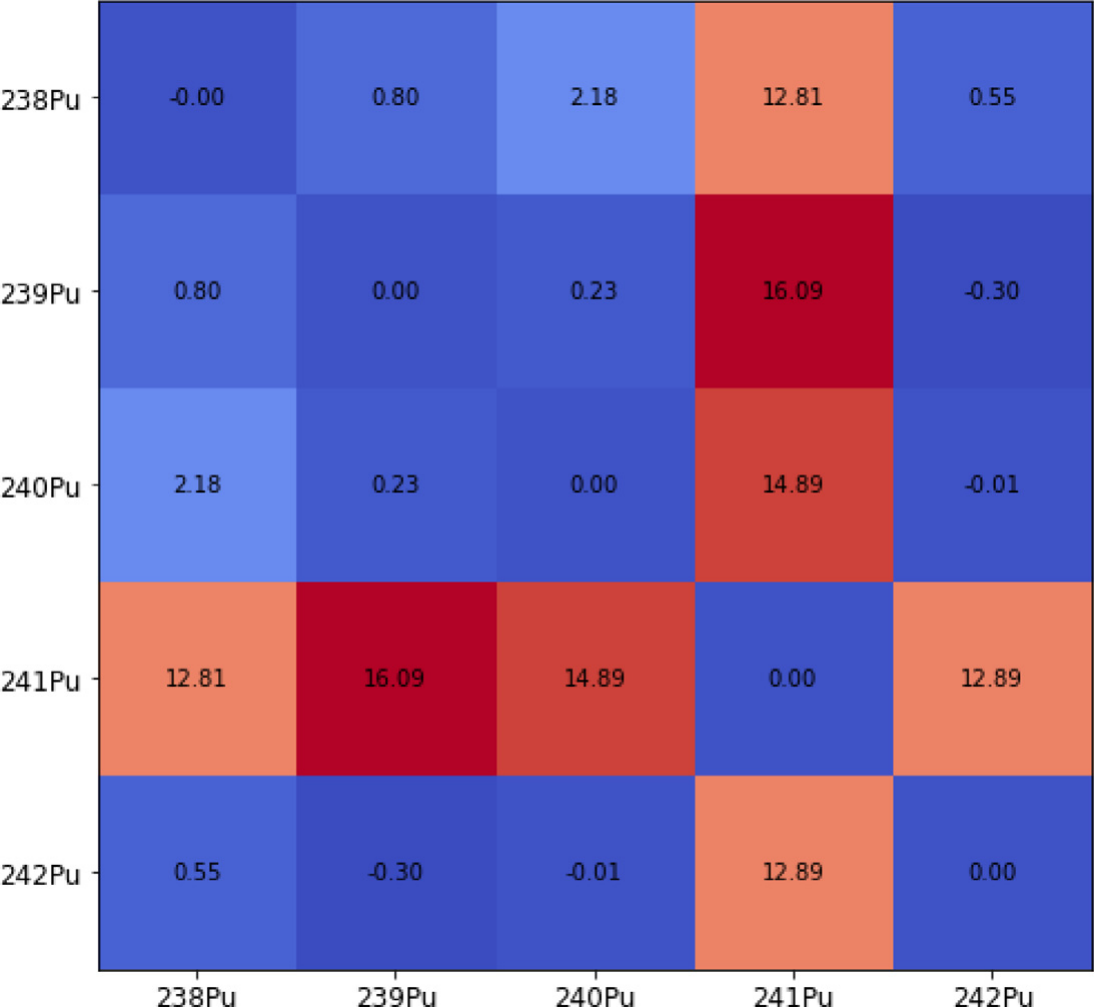
Relative difference in percentage between the final correlation coefficients and the RG reference database.

As a validation measure, the final correlations were compared to those correlations available for Reactor-Grade (RG) plutonium in Europe obtained from [15]. These correlations are coming from the study of real fuel assemblies (FAs): 666 PWRs MOX FAs and 376 Boiling Water Reactors (BWRs) MOX FAs. In Fig. 2, the relative difference from the database sampling with respect to the reference can be observed.

**Fig. 3:**
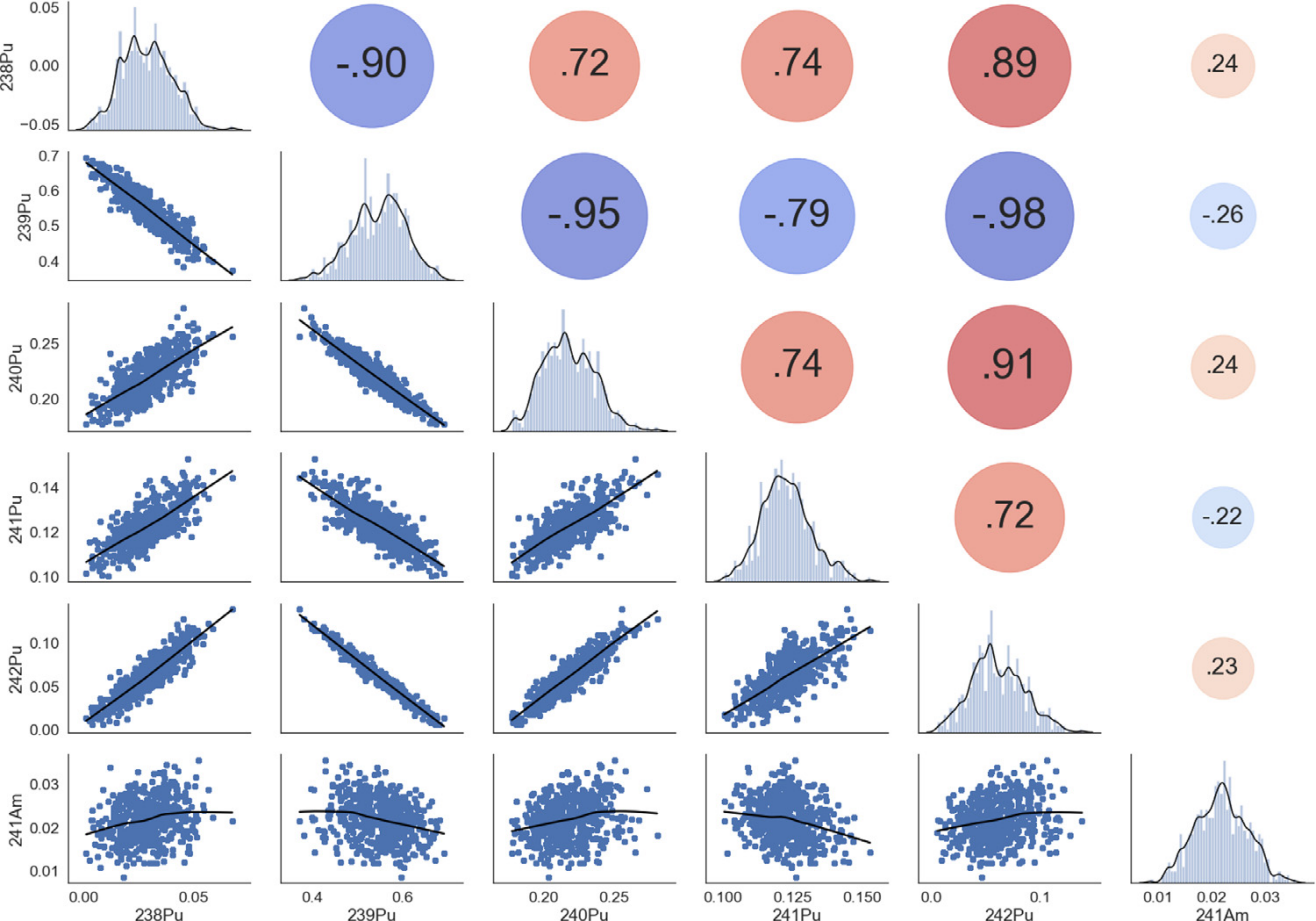
Final plutonium vector composition with its correlation coefficients for the design matrix.

There is a significant discrepancy in the ^241^Pu correlations. Moreover, the RG reference database was made considering different MOX fuel assemblies sampled at different cooling times which can lead to differences in ^241^Am buildup. Additionally, the fuel assemblies recorded in the database were for different reactor technologies, such as BWRs, where the flux is not so homogenously distributed, which may cause some changes in the plutonium breeding. A comprehensive view of the final sample distribution can be observed in Fig. 3.

The ^241^Pu/^241^Am correlation observed (-0.22) reflects the transformation of ^241^Pu to ^241^Am reached on average by sampling the age of the MOX fuel, the correlation found a posteriori in the calculated database for the masses of ^241^Pu/^241^Am was about 0.78.

Once these 500 samples were obtained, they were scaled according to the initial plutonium content. Table 3 shows the steps of each design variable that are part of the design matrix.

**Table 3:** Design space sampling.

Variable	Range/Steps	Sampling points
Rod Linear Power (W/cm)	36.56,73.12, 109.68, 146.24, 182.80 and 219.36	6
Burnup (MWd/kg_HM_)	5 to 150 in steps of 5	30
Initial Plutonium Content (%)	10 to 31 in steps of 3	8
Decay Time (years)	1, 5, 10, 30, 100, 1000, 10 000, 100 000, 1 000 000	9
Pu vector	500 samples from correlations	500

While the regular interval sampling of variables like POWER, BU, and IPC allows for straightforward interpolation, the MVN sampling method provides a more complex, probabilistic representation of the plutonium vector. Thus, the two approaches—regular interval sampling and MVN sampling—are complementary. The first ensures complete coverage of operational and compositional ranges, while the second introduces a realistic representation of the plutonium isotopic distribution based on real-world data.

### 4.2 Methods

Depletion calculations in Serpent2 [5] are performed by successive runs of inputs with the desired material compositions and parameters setup for a series of burnup and decay steps, i.e., the number of inputs to be run corresponds to the number of power, Pu vector, and initial plutonium content sampling points (6 × 500 × 8= 24,000 files).

**Fig. 4:**
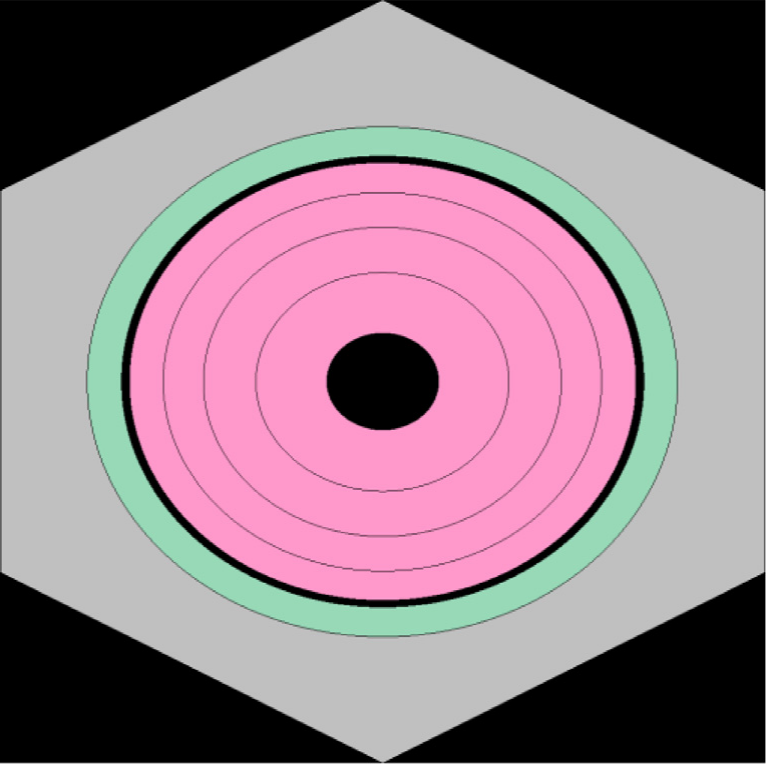
Cross-sectional view of the model's lattice. Depicted in black, void regions, in green the cladding, in grey the coolant and in pink the fuel. Four depletion zones can be observed in the fuel.

The geometry of the model is based on a pin cell containing a cylindrical fuel rod within a hexagonal lattice, which comprises both the fuel pellet and the cladding. This model, based on specifications from Ref. [16], and is representative of MOX-LFR fuel. A depiction of the geometry can be observed in Fig. 4.

The simplified LFR pin-model employed takes then the different range of compositions for MOX as the input of the material fuel definition (mat fuel). The burnup is set for a constant power according to the normalization of the total power of the reactor for that given volume. The parameters related to the general definition of the geometry and the input can be found in Table 4:

**Table 4:** General information about the Serpent2 model.

Serpent Version	2.2.1
Enrichment	From 10.0% to 31.0% for MOX, in steps of 3%
Burnup	5 to 150 MWd/kg_HM_ in steps of 5 MWd/kg_HM_.(dep butot)
Decay (years)	After irradiation: 1, 5, 10, 30, 100, 1000, 10 000,100 000 and 1 000 000
Neutron Cross Section Library	ENDF/B-VIII.0 [17]
Branching ratio libraries	ENDF/B-VIII.0
Decay and fission yield data libraries	ENDF/B-VIII.0
Time integration method	Constant extrapolation (*set pcc 0*)
Power	36.56,73.12, 109.68, 146.24, 182.80 and 219.36 (*set power*)
**Seed**	1423124306
**Restart file**	*set rfw 1*
**Population**	(*set pop 5000 100 10*)
Number of histories per generation	5000
Number of active generations	100
Number of inactive generations	10
Boundary conditions	Reflective (*set bc 2*)
**Energy deposition model**	Local mode 0
**Geometry definition of the problem**	Hexagonal pin cell. Dimensions in cold conditions (20°C), extracted from Ref. [16] *pin 10* *void 0.1* *MOX 0.45* *void 0.465* *cladding 0.525* *coolant* *surf 1 hexxc 0.0 0.0 0.68* *cell c1 0 fill p1 -s1* *cell c2 0 outside s1*
Pellet inside (hollow) radius (mm)	1.00
Pellet outside radius (mm)	4.50
Cladding inside radius (mm)	4.65
Cladding outside radius (mm)	5.25
Pins lattice pitch (mm)	13.60
**Temperature**	
Fuel temperature (K)	1200.0
Cladding temperature(K)	600.0
Coolant temperature (K)	600.0
**Density**	
Fuel density (g/cm^3^)	D = D(*PuO_2_) IPC/100 +* D(*UO_2_) (1-IPC/100) (IPC in %)*
Density PuO_2_ (g/cm^3^)	*10.7771*
Density UO_2_ (g/cm^3^)	*10.3235*
Cladding density (g/cm^3^)	7.972 (AIM/1 Austenitic Stainless Steel)
Coolant density (g/cm^3^)	10.503

Once the calculations were completed, the Depletion Reader module from SerpentTools [18] was used to extract all the relevant information from the outputs stored in the database. The objects extracted from the Depletion Reader were parsed into a data frame using the Pandas tool, and then exported in Parquet format (*SCKCEN-MOX-LFR-SMR.parquet*).

Additionally, the restart files (*set rfw 1*) generated during the first runs, were used to perform the decay calculations (*set rfr idx {bu_index} {restart_file}.wrk*) at the considered decay steps for burnups ranging from 30 MWd/kg_HM_ to 150 MWd/kg_HM_ in steps of 10 MWd/kg_HM_. Finally, the depletion output files were processed the same way that the calculation with no decay, using SerpentTools and parsed into Parquet format (*SCKCEN-MOX-LFR-SMR-DECAY-BU-{XXX}.parquet*)*.*

**Fig. 5:**
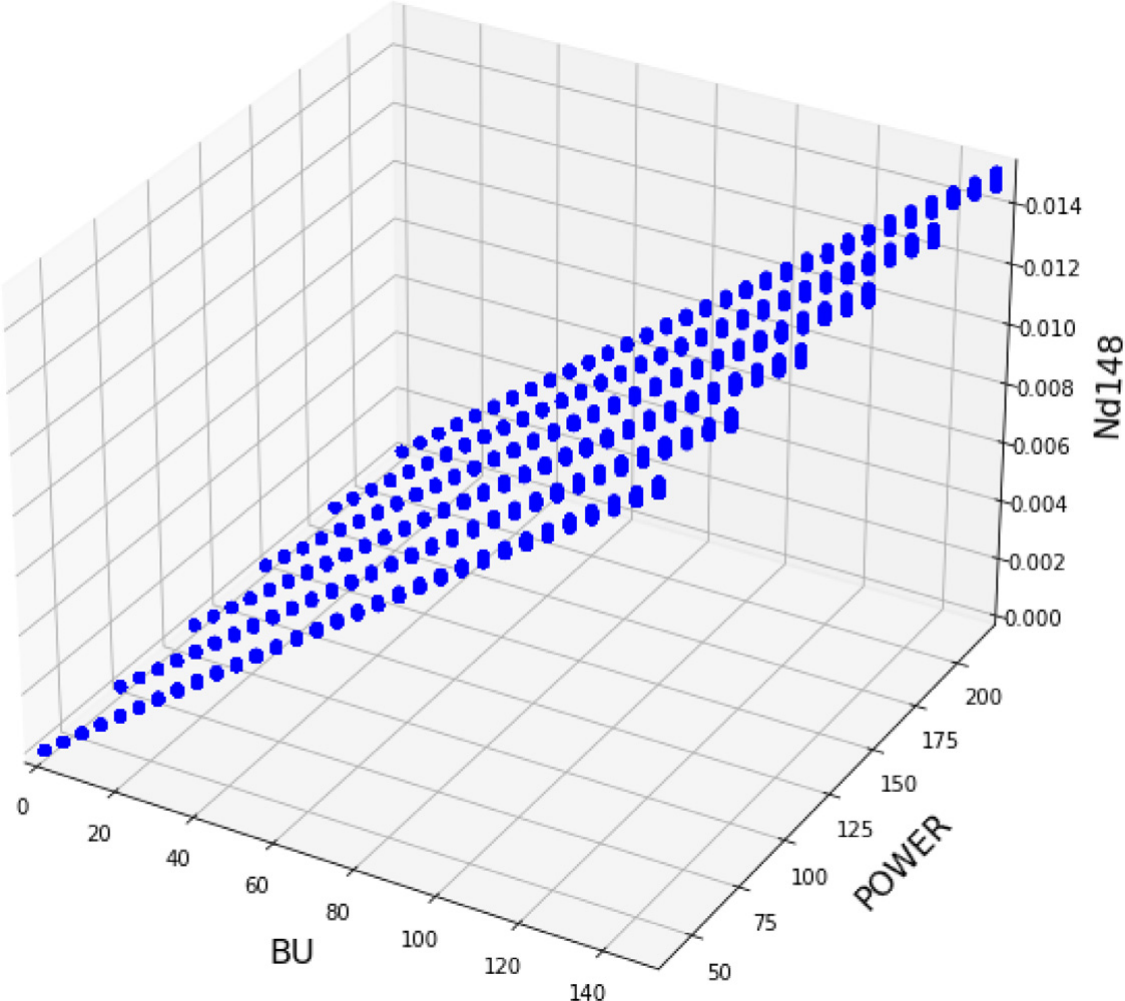
Evolution of ^148^Nd (g/cm^3^) with the burnup (MWd/kg_HM_) and Power (W/cm) for the generated dataset.

As a consistency check, the linearity of the ^148^Nd concentration with burnup was verified in the dataset. ^148^Nd is often referred to as burnup monitor, since it evolves linearly with burnup due to its chemical and neutron-physical properties [19]. As can be seen in Fig. 5, the ^148^Nd concentration behaves as expected.

Neutron emission rate (α,n) term, $s_{\left(\alpha ,n\right)}\left(t\right)$, was subsequently calculated using the Thick Target Yield (TTY) [20] approach:

(1) $$s_{\left(\alpha ,n\right)}\left(t\right)=\sum_{i}N_{i}\left(t\right)\;\lambda_{\alpha ,i}\sum_{j,k}P_{i}\left(E_{\alpha ,k}\right)Y_{l}\left(E_{\alpha ,k}\right)$$

where $N_{i}\left(t\right)$ is the nuclide i number density, $\lambda_{\alpha ,i}$ is the decay constant for α-decay of nuclide i, $P_{i}\left(E_{\alpha ,k}\right)$ is the probability for emission of an α-particle with energy $E_{\alpha ,k}$ from nuclide i undergoing α-decay and $Y_{l}\left(E_{\alpha ,k}\right)$ is the neutron yield for an α-particle with energy $E_{\alpha ,k}$ in the target material l. The quantities $\lambda_{\alpha ,i}$ and $P_{i}\left(E_{\alpha ,k}\right)$ were obtained directly from the nuclear data libraries. The determination of thick target yields $Y_{l}\left(E_{\alpha ,k}\right)$ requires calculating the (α,n) reaction rate during α-particle slowing down in the target material. In this case, the $Y_{l}\left(E_{\alpha ,k}\right)$ values were adopted based on the models available in the SCALE Code System [4].

## Limitations

The observables presented in this dataset have been calculated with low power densities adapted to those to be expected in SMRs; thus trying to extrapolate for greater scale concepts could yield incorrect results. The data is intended to be used only as qualitative assessments in such scenarios.

Pin cell models are often deemed a strong approximation of reality when considering fast spectrum reactors, due to a larger mean free path for neutrons in fast neutron reactors than in thermal spectrum reactors. We deem the data presented here as accurate enough for fuel cycle backend calculations and should not be used for detailed nuclear waste analysis.

Please note that this database is not intended for use with MOX originating from reprocessed MOX. Observables for such cases may differ, as reprocessed MOX typically contains higher proportions of the heavier isotopes in the plutonium vector. These values have been originated considering MOX originated from PWR UOX SNF reprocessing.

## Ethics Statement

The authors state that they have read and followed the ethical requirements for publication in Data in Brief and confirm that the current work does not involve human subjects, animal experiments, or any data collected from social media platforms.

## CRediT authorship contribution statement

**Victor J. Casas-Molina:** Investigation, Methodology, Software, Formal analysis, Data curation, Resources, Writing – original draft. **Pablo Romojaro:** Conceptualization, Project administration, Supervision, Validation, Software, Formal analysis, Writing – original draft, Writing – review & editing. **Gwennaelle Nourry:** Validation, Software. **Gert Van den Eynde:** Conceptualization, Project administration, Supervision, Writing – review & editing. **Luca Fiorito:** Conceptualization, Formal analysis, Writing – review & editing. **Ivan Merino-Rodríguez:** Writing – review & editing. **Tom Dhaene:** Supervision, Writing – review & editing. **Ivo Couckuyt:** Resources, Supervision, Writing – review & editing.

## Data Availability

Mendeley DataObservables of MOX spent fuel simulated in Serpent2 for LFR-SMR (Original data).

## Footnotes

1 That is, plutonium plus americium content prior to irradiation. Depletion is a time-dependent problem, hence, plutonium content evolves with time and initial is used as input.

2 Reprocessing refers to the process of separating spent nuclear fuel into nuclear materials that may be recycled for use in new fuel.
